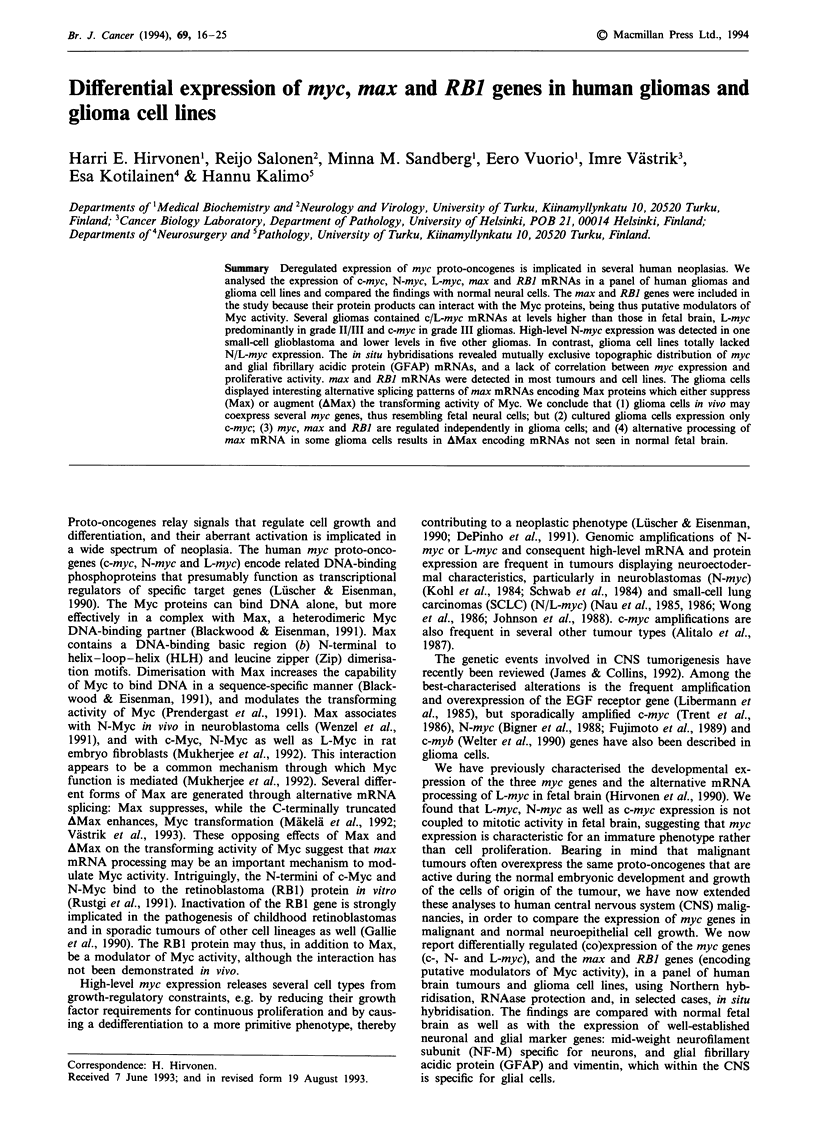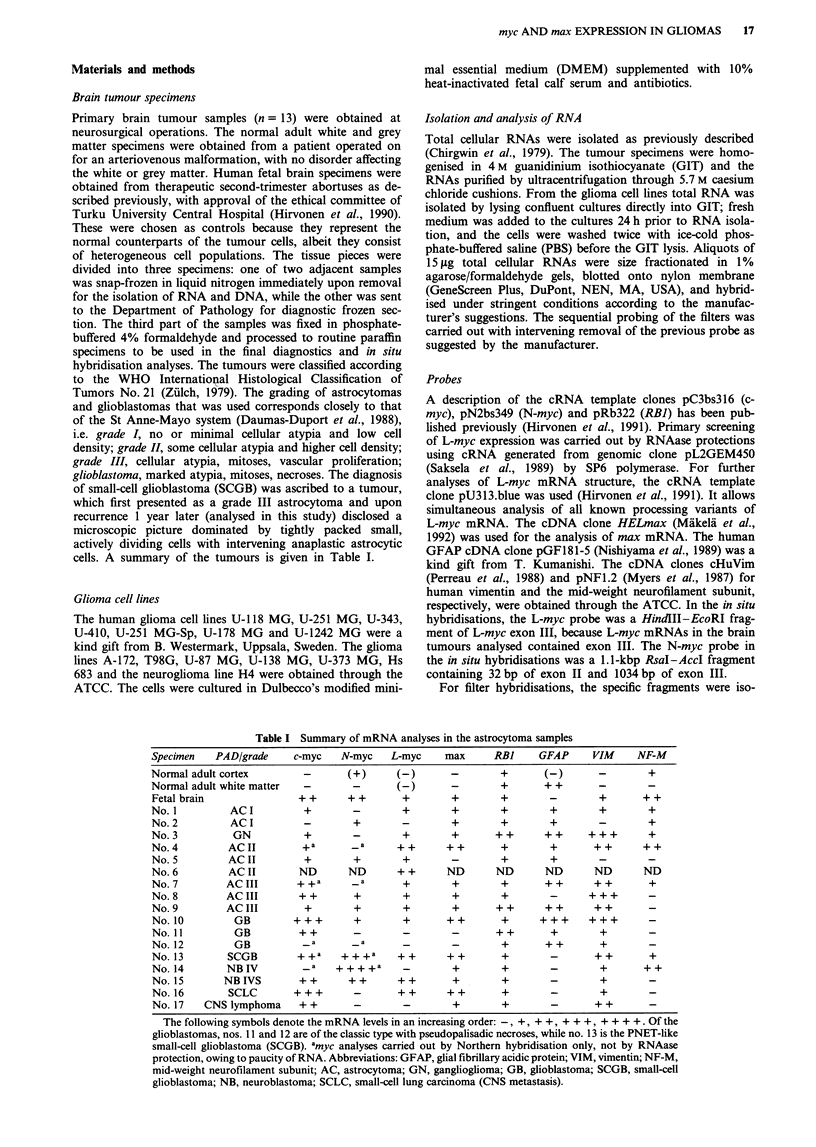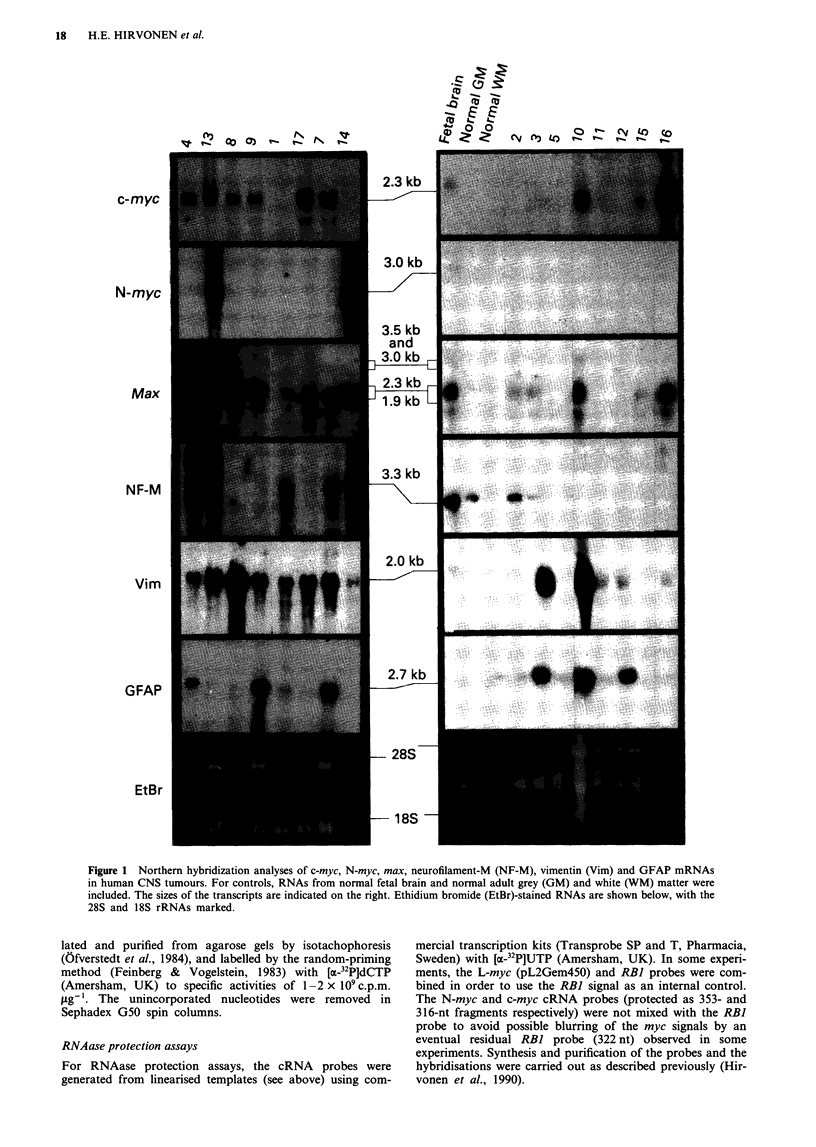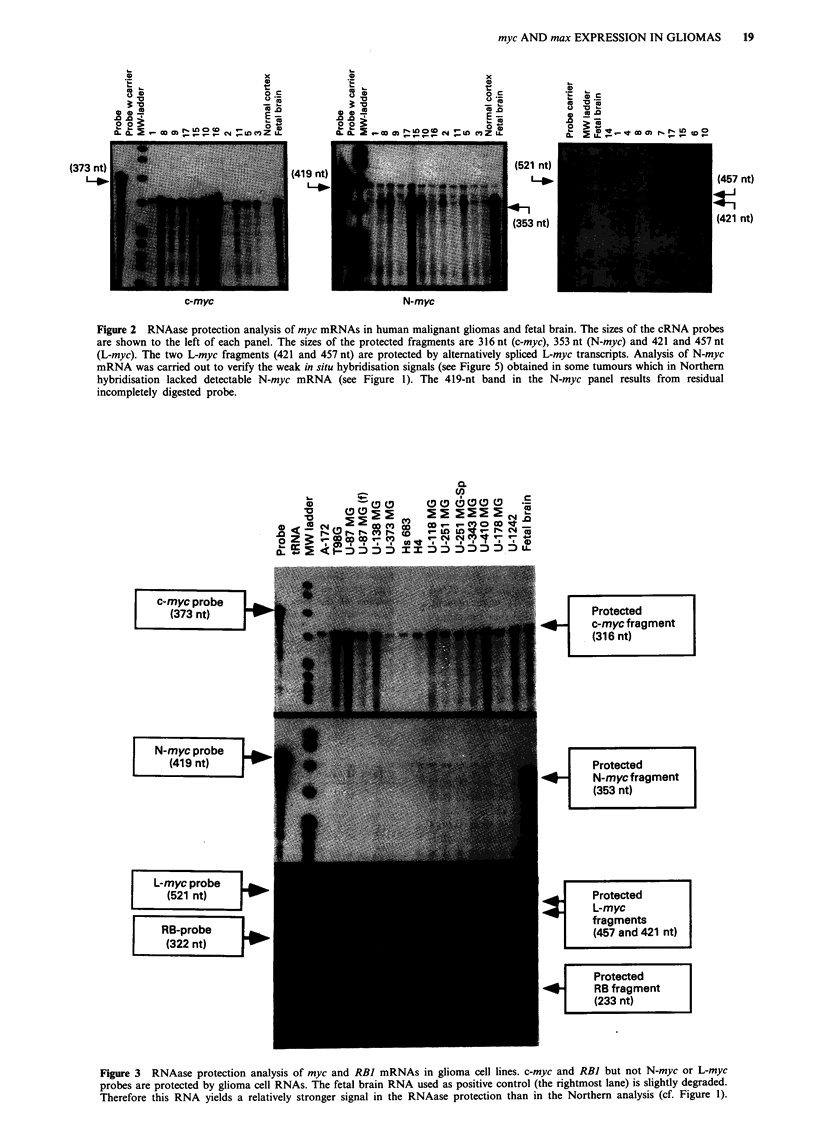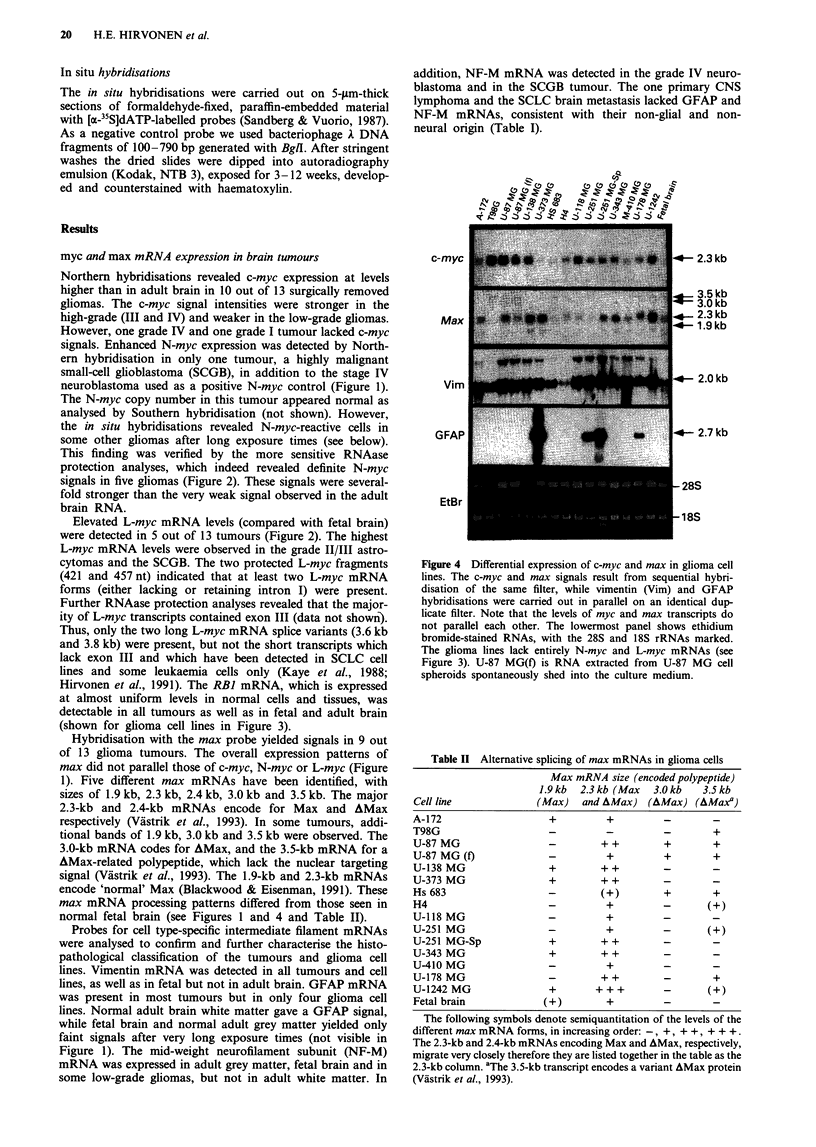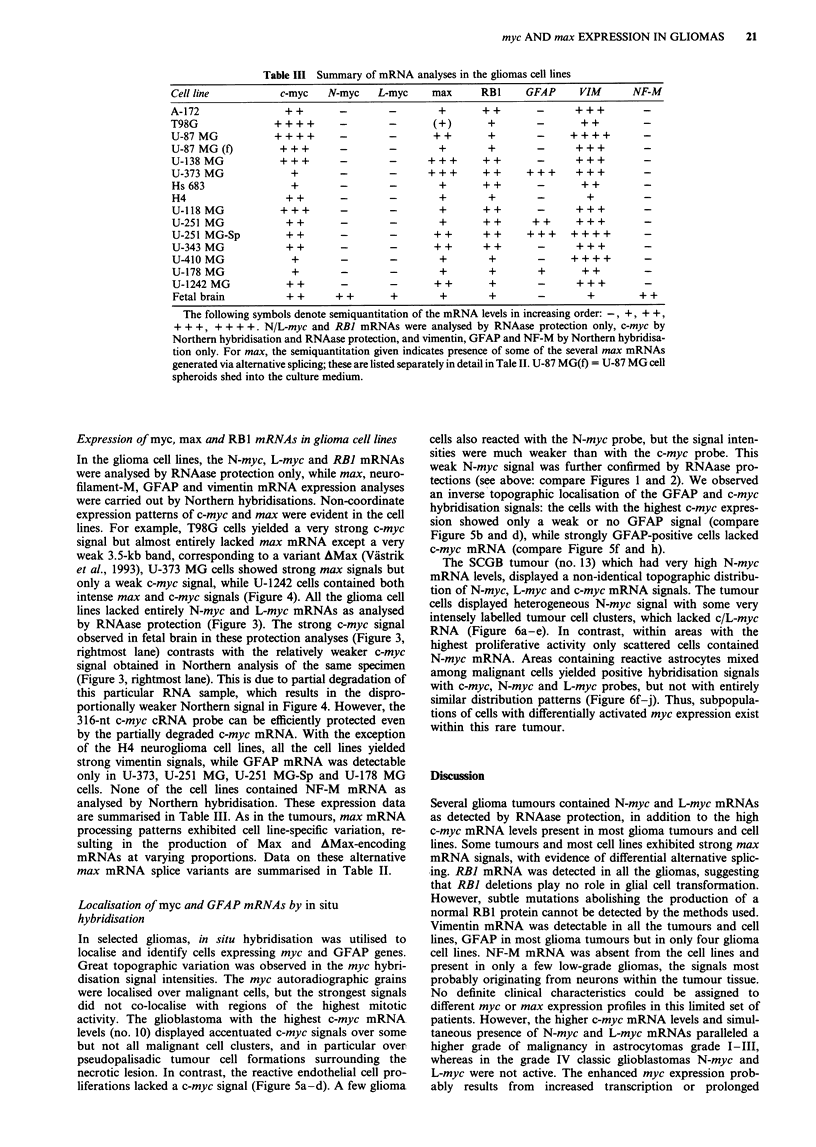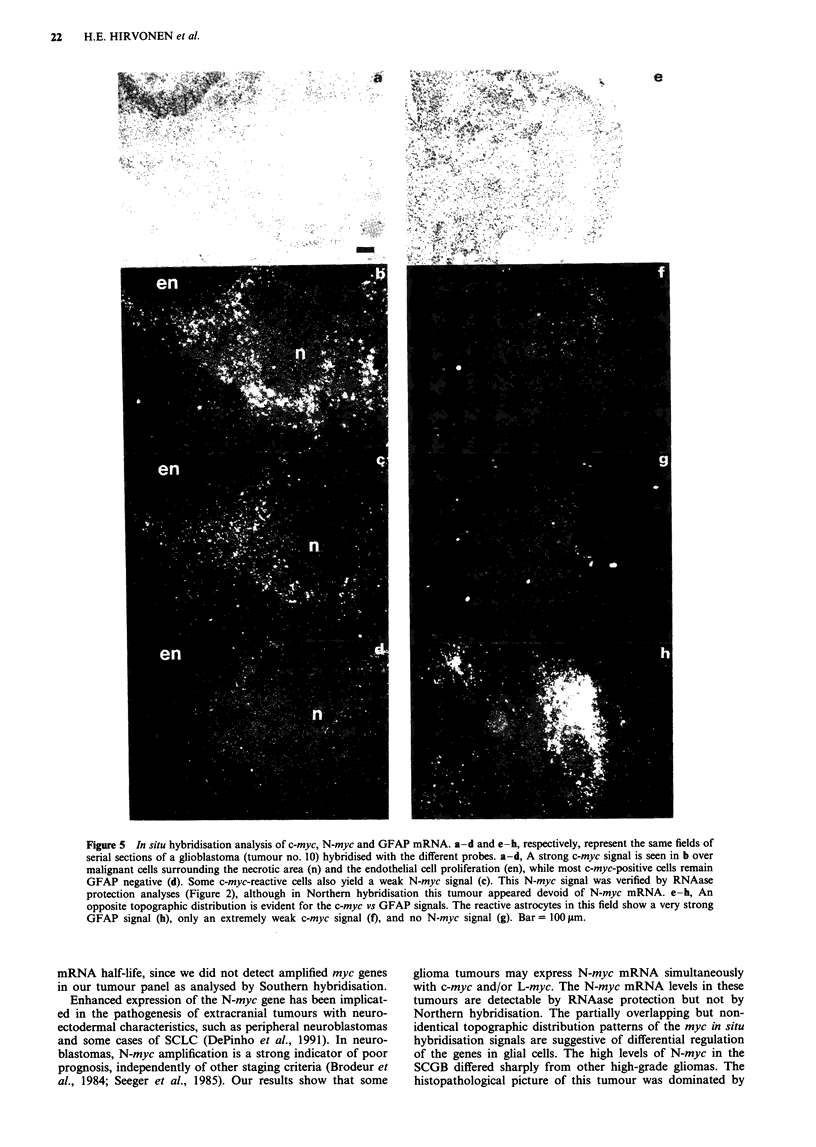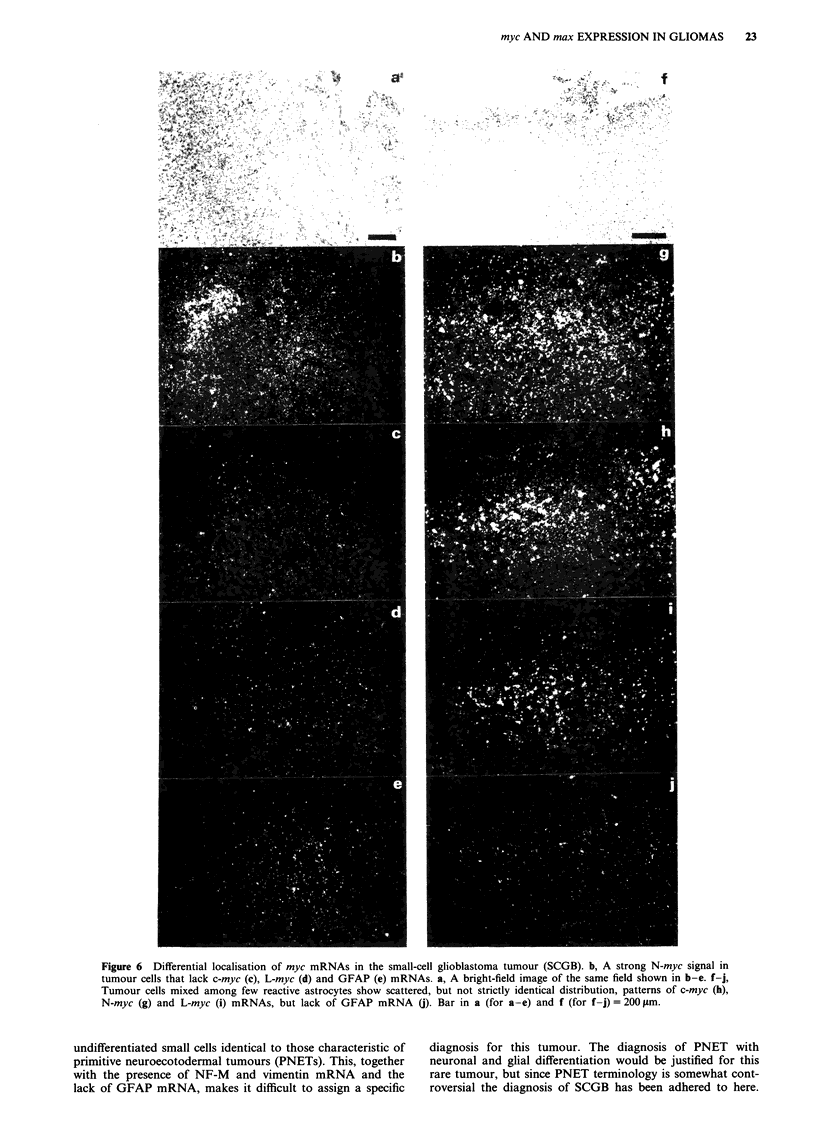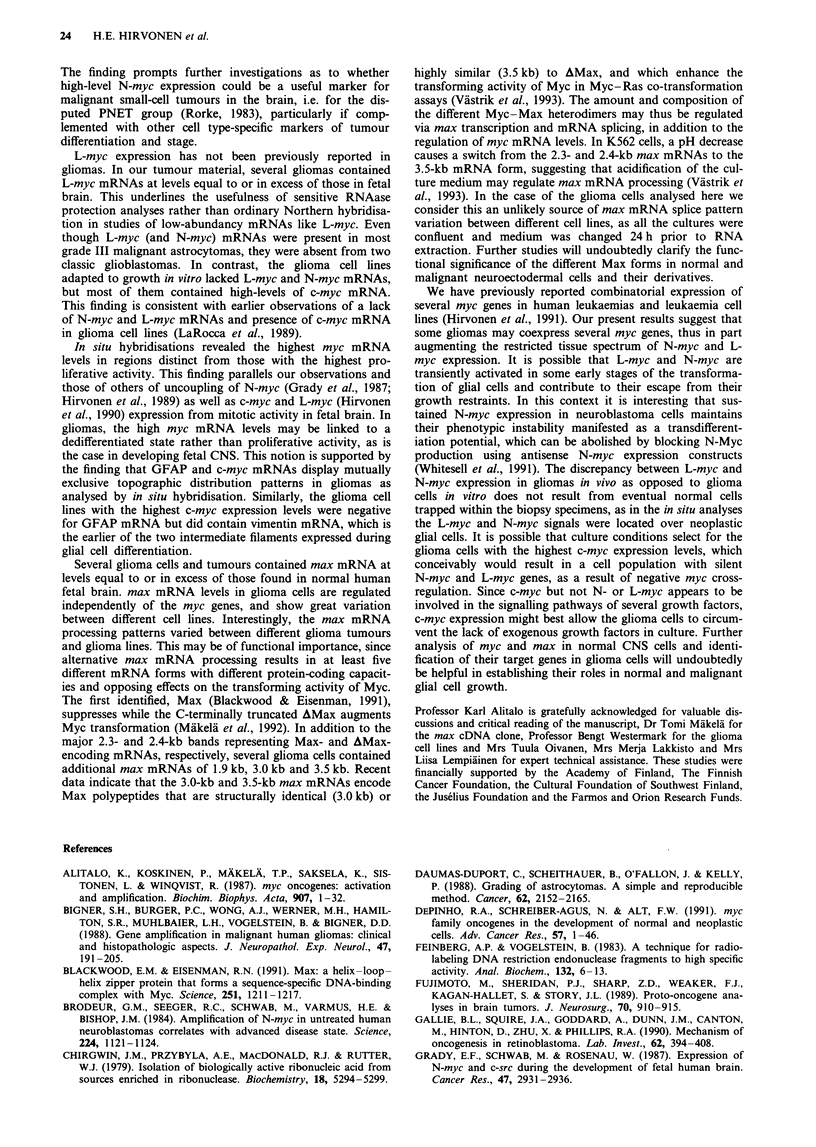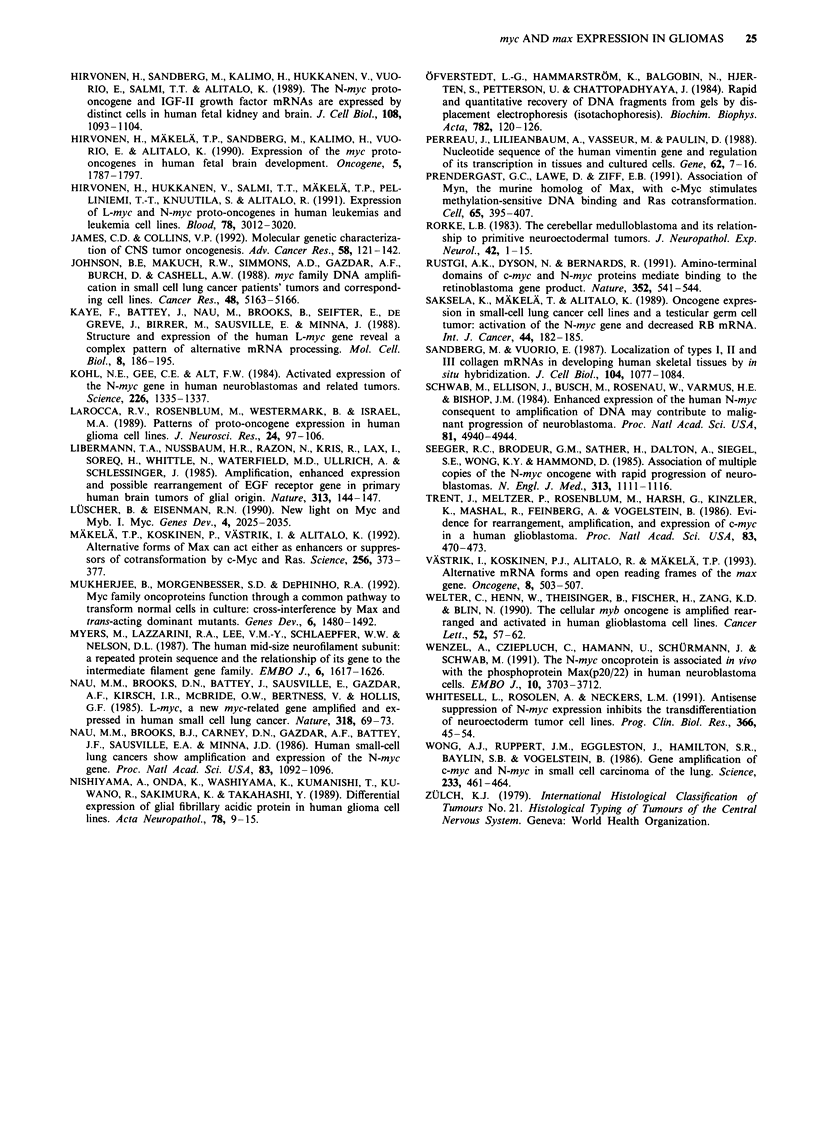# Differential expression of myc, max and RB1 genes in human gliomas and glioma cell lines.

**DOI:** 10.1038/bjc.1994.3

**Published:** 1994-01

**Authors:** H. E. Hirvonen, R. Salonen, M. M. Sandberg, E. Vuorio, I. Västrik, E. Kotilainen, H. Kalimo

**Affiliations:** Department of Medical Biochemistry, University of Turku, Finland.

## Abstract

**Images:**


*Br. J. Cancer* (1994), **69**, 16–25 © Macmillan Press Ltd., 1994

### Differential expression of *myc*, *max* and *RB1* genes in human gliomas and glioma cell lines

Harri E. Hirvonen[1], Reijo Salonen[2], Minna M. Sandberg[1], Eero Vuorio[1], Imre Västrik[3], Esa Kotilainen[4] & Hannu Kalimo[5]

*Departments of [1]Medical Biochemistry and [2]Neurology and Virology, University of Turku, Kiinamyllynkatu 10, 20520 Turku, Finland; [3]Cancer Biology Laboratory, Department of Pathology, University of Helsinki, POB 21, 00014 Helsinki, Finland; Departments of [4]Neurosurgery and [5]Pathology, University of Turku, Kiinamyllynkatu 10, 20520 Turku, Finland.*

**Summary** Deregulated expression of *myc* proto-oncogenes is implicated in several human neoplasias. We analysed the expression of c-*myc*, N-*myc*, L-*myc*, *max* and *RB1* mRNAs in a panel of human gliomas and glioma cell lines and compared the findings with normal neural cells. The *max* and *RB1* genes were included in the study because their protein products can interact with the Myc proteins, being thus putative modulators of Myc activity. Several gliomas contained c/L-*myc* mRNAs at levels higher than those in fetal brain, L-*myc* predominantly in grade II/III and c-*myc* in grade III gliomas. High-level N-*myc* expression was detected in one small-cell glioblastoma and lower levels in five other gliomas. In contrast, glioma cell lines totally lacked N/L-*myc* expression. The *in situ* hybridisations revealed mutually exclusive topographic distribution of *myc* and glial fibrillary acidic protein (GFAP) mRNAs, and a lack of correlation between *myc* expression and proliferative activity. *max* and *RB1* mRNAs were detected in most tumours and cell lines. The glioma cells displayed interesting alternative splicing patterns of *max* mRNAs encoding Max proteins which either suppress (Max) or augment (ΔMax) the transforming activity of Myc. We conclude that (1) glioma cells *in vivo* may coexpress several *myc* genes, thus resembling fetal neural cells; but (2) cultured glioma cells expression only c-*myc*; (3) *myc*, *max* and *RB1* are regulated independently in glioma cells; and (4) alternative processing of *max* mRNA in some glioma cells results in ΔMax encoding mRNAs not seen in normal fetal brain.

Proto-oncogenes relay signals that regulate cell growth and differentiation, and their aberrant activation is implicated in a wide spectrum of neoplasia. The human *myc* proto-oncogenes (c-*myc*, N-*myc* and L-*myc*) encode related DNA-binding phosphoproteins that presumably function as transcriptional regulators of specific target genes (Lüscher & Eisenman, 1990). The Myc proteins can bind DNA alone, but more effectively in a complex with Max, a heterodimeric Myc DNA-binding partner (Blackwood & Eisenman, 1991). Max contains a DNA-binding basic region (*b*) N-terminal to helix–loop–helix (HLH) and leucine zipper (Zip) dimerisation motifs. Dimerisation with Max increases the capability of Myc to bind DNA in a sequence-specific manner (Blackwood & Eisenman, 1991), and modulates the transforming activity of Myc (Prendergast *et al.*, 1991). Max associates with N-Myc *in vivo* in neuroblastoma cells (Wenzel *et al.*, 1991), and with c-Myc, N-Myc as well as L-Myc in rat embryo fibroblasts (Mukherjee *et al.*, 1992). This interaction appears to be a common mechanism through which Myc function is mediated (Mukherjee *et al.*, 1992). Several different forms of Max are generated through alternative mRNA splicing: Max suppresses, while the C-terminally truncated ΔMax enhances, Myc transformation (Mäkelä *et al.*, 1992; Västrik *et al.*, 1993). These opposing effects of Max and ΔMax on the transforming activity of Myc suggest that *max* mRNA processing may be an important mechanism to modulate Myc activity. Intriguingly, the N-termini of c-Myc and N-Myc bind to the retinoblastoma (RB1) protein *in vitro* (Rustgi *et al.*, 1991). Inactivation of the RB1 gene is strongly implicated in the pathogenesis of childhood retinoblastomas and in sporadic tumours of other cell lineages as well (Gallie *et al.*, 1990). The RB1 protein may thus, in addition to Max, be a modulator of Myc activity, although the interaction has not been demonstrated *in vivo*.

High-level *myc* expression releases several cell types from growth-regulatory constraints, e.g. by reducing their growth factor requirements for continuous proliferation and by causing a dedifferentiation to a more primitive phenotype, thereby contributing to a neoplastic phenotype (Lüscher & Eisenman, 1990; DePinho *et al.*, 1991). Genomic amplifications of N-*myc* or L-*myc* and consequent high-level mRNA and protein expression are frequent in tumours displaying neuroectodermal characteristics, particularly in neuroblastomas (N-*myc*) (Kohl *et al.*, 1984; Schwab *et al.*, 1984) and small-cell lung carcinomas (SCLC) (N/L-*myc*) (Nau *et al.*, 1985, 1986; Wong *et al.*, 1986; Johnson *et al.*, 1988). c-*myc* amplifications are also frequent in several other tumour types (Alitalo *et al.*, 1987).

The genetic events involved in CNS tumorigenesis have recently been reviewed (James & Collins, 1992). Among the best-characterised alterations is the frequent amplification and overexpression of the EGF receptor gene (Libermann *et al.*, 1985), but sporadically amplified c-*myc* (Trent *et al.*, 1986), N-*myc* (Bigner *et al.*, 1988; Fujimoto *et al.*, 1989) and c-*myb* (Welter *et al.*, 1990) genes have also been described in glioma cells.

We have previously characterised the developmental expression of the three *myc* genes and the alternative mRNA processing of L-*myc* in fetal brain (Hirvonen *et al.*, 1990). We found that L-*myc*, N-*myc* as well as c-*myc* expression is not coupled to mitotic activity in fetal brain, suggesting that *myc* expression is characteristic for an immature phenotype rather than cell proliferation. Bearing in mind that malignant tumours often overexpress the same proto-oncogenes that are active during the normal embryonic development and growth of the cells of origin of the tumour, we have now extended these analyses to human central nervous system (CNS) malignancies, in order to compare the expression of *myc* genes in malignant and normal neuroepithelial cell growth. We now report differentially regulated (co)expression of the *myc* genes (c-, N- and L-*myc*), and the *max* and *RB1* genes (encoding putative modulators of Myc activity), in a panel of human brain tumours and glioma cell lines, using Northern hybridisation, RNAase protection and, in selected cases, *in situ* hybridisation. The findings are compared with normal fetal brain as well as with the expression of well-established neuronal and glial marker genes: mid-weight neurofilament subunit (NF-M) specific for neurons, and glial fibrillary acidic protein (GFAP) and vimentin, which within the CNS is specific for glial cells.

Correspondence: H. Hirvonen.
Received 7 June 1993; and in revised form 19 August 1993.

### Materials and methods

### *Brain tumour specimens*

Primary brain tumour samples ($n = 13$) were obtained at neurosurgical operations. The normal adult white and grey matter specimens were obtained from a patient operated on for an arteriovenous malformation, with no disorder affecting the white or grey matter. Human fetal brain specimens were obtained from therapeutic second-trimester abortuses as described previously, with approval of the ethical committee of Turku University Central Hospital (Hirvonen *et al.*, 1990). These were chosen as controls because they represent the normal counterparts of the tumour cells, albeit they consist of heterogeneous cell populations. The tissue pieces were divided into three specimens: one of two adjacent samples was snap-frozen in liquid nitrogen immediately upon removal for the isolation of RNA and DNA, while the other was sent to the Department of Pathology for diagnostic frozen section. The third part of the samples was fixed in phosphate-buffered 4% formaldehyde and processed to routine paraffin specimens to be used in the final diagnostics and *in situ* hybridisation analyses. The tumours were classified according to the WHO International Histological Classification of Tumors No. 21 (Zülch, 1979). The grading of astrocytomas and glioblastomas that was used corresponds closely to that of the St Anne-Mayo system (Daumas-Duport *et al.*, 1988), i.e. *grade I*, no or minimal cellular atypia and low cell density; *grade II*, some cellular atypia and higher cell density; *grade III*, cellular atypia, mitoses, vascular proliferation; *glioblastoma*, marked atypia, mitoses, necroses. The diagnosis of small-cell glioblastoma (SCGB) was ascribed to a tumour, which first presented as a grade III astrocytoma and upon recurrence 1 year later (analysed in this study) disclosed a microscopic picture dominated by tightly packed small, actively dividing cells with intervening anaplastic astrocytic cells. A summary of the tumours is given in Table I.

### *Glioma cell lines*

The human glioma cell lines U-118 MG, U-251 MG, U-343, U-410, U-251 MG-Sp, U-178 MG and U-1242 MG were a kind gift from B. Westermark, Uppsala, Sweden. The glioma lines A-172, T98G, U-87 MG, U-138 MG, U-373 MG, Hs 683 and the neuroglioma line H4 were obtained through the ATCC. The cells were cultured in Dulbecco's modified minimal essential medium (DMEM) supplemented with 10% heat-inactivated fetal calf serum and antibiotics.

### *Isolation and analysis of RNA*

Total cellular RNAs were isolated as previously described (Chirgwin *et al.*, 1979). The tumour specimens were homogenised in 4 M guanidinium isothiocyanate (GIT) and the RNAs purified by ultracentrifugation through 5.7 M caesium chloride cushions. From the glioma cell lines total RNA was isolated by lysing confluent cultures directly into GIT; fresh medium was added to the cultures 24 h prior to RNA isolation, and the cells were washed twice with ice-cold phosphate-buffered saline (PBS) before the GIT lysis. Aliquots of 15 μg total cellular RNAs were size fractionated in 1% agarose/formaldehyde gels, blotted onto nylon membrane (GeneScreen Plus, DuPont, NEN, MA, USA), and hybridised under stringent conditions according to the manufacturer's suggestions. The sequential probing of the filters was carried out with intervening removal of the previous probe as suggested by the manufacturer.

### *Probes*

A description of the cRNA template clones pC3bs316 (c-*myc*), pN2bs349 (N-*myc*) and pRb322 (*RB1*) has been published previously (Hirvonen *et al.*, 1991). Primary screening of L-*myc* expression was carried out by RNAase protections using cRNA generated from genomic clone pL2GEM450 (Saksela *et al.*, 1989) by SP6 polymerase. For further analyses of L-*myc* mRNA structure, the cRNA template clone pU313.blue was used (Hirvonen *et al.*, 1991). It allows simultaneous analysis of all known processing variants of L-*myc* mRNA. The cDNA clone *HELmax* (Mäkelä *et al.*, 1992) was used for the analysis of *max* mRNA. The human GFAP cDNA clone pGF181-5 (Nishiyama *et al.*, 1989) was a kind gift from T. Kumanishi. The cDNA clones cHuVim (Perreau *et al.*, 1988) and pNF1.2 (Myers *et al.*, 1987) for human vimentin and the mid-weight neurofilament subunit, respectively, were obtained through the ATCC. In the *in situ* hybridisations, the L-*myc* probe was a *Hin*dIII–*Eco*RI fragment of L-*myc* exon III, because L-*myc* mRNAs in the brain tumours analysed contained exon III. The N-*myc* probe in the *in situ* hybridisations was a 1.1-kbp *Rsa*I–*Acc*I fragment containing 32 bp of exon II and 1034 bp of exon III.

For filter hybridisations, the specific fragments were iso-

**Table I** Summary of mRNA analyses in the astrocytoma samples

| *Specimen* | *PAD/grade* | *c*-myc | *N*-myc | *L*-myc | max | *RB1* | *GFAP* | *VIM* | *NF-M* |
|---|---|---|---|---|---|---|---|---|---|
| Normal adult cortex | | – | (+) | (–) | – | + | (–) | – | + |
| Normal adult white matter | | – | – | (–) | – | + | + + | – | – |
| Fetal brain | | + + | + + | + | + | + | – | + | + + |
| No. 1 | AC I | + | – | + | + | + | + | + | + |
| No. 2 | AC I | – | + | – | + | + | + | – | + |
| No. 3 | GN | + | – | + | + | + + | + + | + + + | + |
| No. 4 | AC II | +[a] | –[a] | + + | + + | + | + | + + | + + |
| No. 5 | AC II | + | + | + | – | + | + | – | – |
| No. 6 | AC II | ND | ND | + + | ND | ND | ND | ND | ND |
| No. 7 | AC III | + +[a] | –[a] | + | + | + | + + | + + | + |
| No. 8 | AC III | + + | + | + | + | + | – | + + + | – |
| No. 9 | AC III | + | + | + | + | + + | + + | + + | – |
| No. 10 | GB | + + + | + | + | + + | + | + + + | + + + | – |
| No. 11 | GB | + + | – | – | – | + + | + | + | – |
| No. 12 | GB | –[a] | –[a] | – | – | + | + + | + | – |
| No. 13 | SCGB | + +[a] | + + +[a] | + + | + + | + | – | + + | + |
| No. 14 | NB IV | –[a] | + + + +[a] | – | + | + | – | + | + + |
| No. 15 | NB IVS | + + | + + | + + | + | + | – | + | – |
| No. 16 | SCLC | + + + | – | + + | + + | + | – | + | – |
| No. 17 | CNS lymphoma | + + | – | – | + | + | – | + + | – |

The following symbols denote the mRNA levels in an increasing order: –, +, + +, + + +, + + + +. Of the glioblastomas, nos. 11 and 12 are of the classic type with pseudopalisadic necroses, while no. 13 is the PNET-like small-cell glioblastoma (SCGB). [a]*myc* analyses carried out by Northern hybridisation only, not by RNAase protection, owing to paucity of RNA. Abbreviations: GFAP, glial fibrillary acidic protein; VIM, vimentin; NF-M, mid-weight neurofilament subunit; AC, astrocytoma; GN, ganglioglioma; GB, glioblastoma; SCGB, small-cell glioblastoma; NB, neuroblastoma; SCLC, small-cell lung carcinoma (CNS metastasis).

**Figure 1** Northern hybridization analyses of c-*myc*, N-*myc*, *max*, neurofilament-M (NF-M), vimentin (Vim) and GFAP mRNAs in human CNS tumours. For controls, RNAs from normal fetal brain and normal adult grey (GM) and white (WM) matter were included. The sizes of the transcripts are indicated on the right. Ethidium bromide (EtBr)-stained RNAs are shown below, with the 28S and 18S rRNAs marked.

lated and purified from agarose gels by isotachophoresis (Öfverstedt *et al.*, 1984), and labelled by the random-priming method (Feinberg & Vogelstein, 1983) with [$\alpha$-$^{32}$P]dCTP (Amersham, UK) to specific activities of $1-2 \times 10^9$ c.p.m. $\mu g^{-1}$. The unincorporated nucleotides were removed in Sephadex G50 spin columns.

*RNAase protection assays*

For RNAase protection assays, the cRNA probes were generated from linearised templates (see above) using commercial transcription kits (Transprobe SP and T, Pharmacia, Sweden) with [$\alpha$-$^{32}$P]UTP (Amersham, UK). In some experiments, the L-*myc* (pL2Gem450) and *RB1* probes were combined in order to use the *RB1* signal as an internal control. The N-*myc* and c-*myc* cRNA probes (protected as 353- and 316-nt fragments respectively) were not mixed with the *RB1* probe to avoid possible blurring of the *myc* signals by an eventual residual *RB1* probe (322 nt) observed in some experiments. Synthesis and purification of the probes and the hybridisations were carried out as described previously (Hirvonen *et al.*, 1990).

**Figure 2** RNAase protection analysis of *myc* mRNAs in human malignant gliomas and fetal brain. The sizes of the cRNA probes are shown to the left of each panel. The sizes of the protected fragments are 316 nt (c-*myc*), 353 nt (N-*myc*) and 421 and 457 nt (L-*myc*). The two L-*myc* fragments (421 and 457 nt) are protected by alternatively spliced L-*myc* transcripts. Analysis of N-*myc* mRNA was carried out to verify the weak *in situ* hybridisation signals (see Figure 5) obtained in some tumours which in Northern hybridisation lacked detectable N-*myc* mRNA (see Figure 1). The 419-nt band in the N-*myc* panel results from residual incompletely digested probe.

**Figure 3** RNAase protection analysis of *myc* and *RB1* mRNAs in glioma cell lines. c-*myc* and *RB1* but not N-*myc* or L-*myc* probes are protected by glioma cell RNAs. The fetal brain RNA used as positive control (the rightmost lane) is slightly degraded. Therefore this RNA yields a relatively stronger signal in the RNAase protection than in the Northern analysis (cf. Figure 1).

### In situ *hybridisations*

The *in situ* hybridisations were carried out on 5-μm-thick sections of formaldehyde-fixed, paraffin-embedded material with [α-$^{35}$S]dATP-labelled probes (Sandberg & Vuorio, 1987). As a negative control probe we used bacteriophage λ DNA fragments of 100–790 bp generated with *BglI*. After stringent washes the dried slides were dipped into autoradiography emulsion (Kodak, NTB 3), exposed for 3–12 weeks, developed and counterstained with haematoxylin.

### Results

### myc *and* max *mRNA expression in brain tumours*

Northern hybridisations revealed c-*myc* expression at levels higher than in adult brain in 10 out of 13 surgically removed gliomas. The c-*myc* signal intensities were stronger in the high-grade (III and IV) and weaker in the low-grade gliomas. However, one grade IV and one grade I tumour lacked c-*myc* signals. Enhanced N-*myc* expression was detected by Northern hybridisation in only one tumour, a highly malignant small-cell glioblastoma (SCGB), in addition to the stage IV neuroblastoma used as a positive N-*myc* control (Figure 1). The N-*myc* copy number in this tumour appeared normal as analysed by Southern hybridisation (not shown). However, the *in situ* hybridisations revealed N-*myc*-reactive cells in some other gliomas after long exposure times (see below). This finding was verified by the more sensitive RNAase protection analyses, which indeed revealed definite N-*myc* signals in five gliomas (Figure 2). These signals were several-fold stronger than the very weak signal observed in the adult brain RNA.

Elevated L-*myc* mRNA levels (compared with fetal brain) were detected in 5 out of 13 tumours (Figure 2). The highest L-*myc* mRNA levels were observed in the grade II/III astrocytomas and the SCGB. The two protected L-*myc* fragments (421 and 457 nt) indicated that at least two L-*myc* mRNA forms (either lacking or retaining intron I) were present. Further RNAase protection analyses revealed that the majority of L-*myc* transcripts contained exon III (data not shown). Thus, only the two long L-*myc* mRNA splice variants (3.6 kb and 3.8 kb) were present, but not the short transcripts which lack exon III and which have been detected in SCLC cell lines and some leukaemia cells only (Kaye *et al.*, 1988; Hirvonen *et al.*, 1991). The *RB1* mRNA, which is expressed at almost uniform levels in normal cells and tissues, was detectable in all tumours as well as in fetal and adult brain (shown for glioma cell lines in Figure 3).

Hybridisation with the *max* probe yielded signals in 9 out of 13 glioma tumours. The overall expression patterns of *max* did not parallel those of c-*myc*, N-*myc* or L-*myc* (Figure 1). Five different *max* mRNAs have been identified, with sizes of 1.9 kb, 2.3 kb, 2.4 kb, 3.0 kb and 3.5 kb. The major 2.3-kb and 2.4-kb mRNAs encode for Max and ΔMax respectively (Västrik *et al.*, 1993). In some tumours, additional bands of 1.9 kb, 3.0 kb and 3.5 kb were observed. The 3.0-kb mRNA codes for ΔMax, and the 3.5-kb mRNA for a ΔMax-related polypeptide, which lack the nuclear targeting signal (Västrik *et al.*, 1993). The 1.9-kb and 2.3-kb mRNAs encode 'normal' Max (Blackwood & Eisenman, 1991). These *max* mRNA processing patterns differed from those seen in normal fetal brain (see Figures 1 and 4 and Table II).

Probes for cell type-specific intermediate filament mRNAs were analysed to confirm and further characterise the histopathological classification of the tumours and glioma cell lines. Vimentin mRNA was detected in all tumours and cell lines, as well as in fetal but not in adult brain. GFAP mRNA was present in most tumours but in only four glioma cell lines. Normal adult brain white matter gave a GFAP signal, while fetal brain and normal adult grey matter yielded only faint signals after very long exposure times (not visible in Figure 1). The mid-weight neurofilament subunit (NF-M) mRNA was expressed in adult grey matter, fetal brain and in some low-grade gliomas, but not in adult white matter. In addition, NF-M mRNA was detected in the grade IV neuroblastoma and in the SCGB tumour. The one primary CNS lymphoma and the SCLC brain metastasis lacked GFAP and NF-M mRNAs, consistent with their non-glial and non-neural origin (Table I).

**Figure 4** Differential expression of c-*myc* and *max* in glioma cell lines. The c-*myc* and *max* signals result from sequential hybridisation of the same filter, while vimentin (Vim) and GFAP hybridisations were carried out in parallel on an identical duplicate filter. Note that the levels of *myc* and *max* transcripts do not parallel each other. The lowermost panel shows ethidium bromide-stained RNAs, with the 28S and 18S rRNAs marked. The glioma lines lack entirely N-*myc* and L-*myc* mRNAs (see Figure 3). U-87 MG(f) is RNA extracted from U-87 MG cell spheroids spontaneously shed into the culture medium.

**Table II** Alternative splicing of *max* mRNAs in glioma cells

| *Cell line* | *Max mRNA size (encoded polypeptide)* 1.9 kb (Max) | 2.3 kb (Max and ΔMax) | 3.0 kb (ΔMax) | 3.5 kb (ΔMax[a]) |
|---|---|---|---|---|
| A-172 | + | + | – | – |
| T98G | – | – | – | + |
| U-87 MG | – | ++ | + | + |
| U-87 MG (f) | – | + | + | + |
| U-138 MG | + | ++ | – | – |
| U-373 MG | + | ++ | – | – |
| Hs 683 | – | (+) | + | + |
| H4 | – | + | – | (+) |
| U-118 MG | – | + | – | – |
| U-251 MG | – | + | – | (+) |
| U-251 MG-Sp | + | ++ | – | – |
| U-343 MG | + | ++ | – | – |
| U-410 MG | – | + | – | – |
| U-178 MG | – | ++ | – | + |
| U-1242 MG | + | +++ | – | (+) |
| Fetal brain | (+) | + | – | – |

The following symbols denote semiquantitation of the levels of the different *max* mRNA forms, in increasing order: –, +, ++, +++. The 2.3-kb and 2.4-kb mRNAs encoding Max and ΔMax, respectively, migrate very closely therefore they are listed together in the table as the 2.3-kb column. [a]The 3.5-kb transcript encodes a variant ΔMax protein (Västrik *et al.*, 1993).

**Table III** Summary of mRNA analyses in the gliomas cell lines

| *Cell line* | *c*-myc | *N*-myc | *L*-myc | max | RB1 | *GFAP* | *VIM* | *NF-M* |
|---|---|---|---|---|---|---|---|---|
| A-172 | + + | – | – | + | + + | – | + + + | – |
| T98G | + + + + | – | – | (+) | + | – | + + | – |
| U-87 MG | + + + + | – | – | + + | + | – | + + + + | – |
| U-87 MG (f) | + + + | – | – | + | + | – | + + + | – |
| U-138 MG | + + + | – | – | + + + | + + | – | + + + | – |
| U-373 MG | + | – | – | + + + | + + | + + + | + + + | – |
| Hs 683 | + | – | – | + | + + | – | + + | – |
| H4 | + + | – | – | + | + | – | + | – |
| U-118 MG | + + + | – | – | + | + + | – | + + + | – |
| U-251 MG | + + | – | – | + | + + | + + | + + + | – |
| U-251 MG-Sp | + + | – | – | + + | + + | + + + | + + + + | – |
| U-343 MG | + + | – | – | + + | + + | – | + + + | – |
| U-410 MG | + | – | – | + | + | – | + + + + | – |
| U-178 MG | + | – | – | + | + | + | + + | – |
| U-1242 MG | + + | – | – | + + | + | – | + + + | – |
| Fetal brain | + + | + + | + | + | + | – | + | + + |

The following symbols denote semiquantitation of the mRNA levels in increasing order: –, +, + +, + + +, + + + +. N/L-*myc* and *RB1* mRNAs were analysed by RNAase protection only, c-*myc* by Northern hybridisation and RNAase protection, and vimentin, GFAP and NF-M by Northern hybridisation only. For *max*, the semiquantitation given indicates presence of some of the several *max* mRNAs generated via alternative splicing; these are listed separately in detail in Tale II. U-87 MG(f) = U-87 MG cell spheroids shed into the culture medium.

### *Expression of* myc, max *and* RB1 *mRNAs in glioma cell lines*

In the glioma cell lines, the N-*myc*, L-*myc* and *RB1* mRNAs were analysed by RNAase protection only, while *max*, neurofilament-M, GFAP and vimentin mRNA expression analyses were carried out by Northern hybridisations. Non-coordinate expression patterns of c-*myc* and *max* were evident in the cell lines. For example, T98G cells yielded a very strong c-*myc* signal but almost entirely lacked *max* mRNA except a very weak 3.5-kb band, corresponding to a variant ΔMax (Västrik *et al.*, 1993), U-373 MG cells showed strong *max* signals but only a weak c-*myc* signal, while U-1242 cells contained both intense *max* and c-*myc* signals (Figure 4). All the glioma cell lines lacked entirely N-*myc* and L-*myc* mRNAs as analysed by RNAase protection (Figure 3). The strong c-*myc* signal observed in fetal brain in these protection analyses (Figure 3, rightmost lane) contrasts with the relatively weaker c-*myc* signal obtained in Northern analysis of the same specimen (Figure 3, rightmost lane). This is due to partial degradation of this particular RNA sample, which results in the disproportionally weaker Northern signal in Figure 4. However, the 316-nt c-*myc* cRNA probe can be efficiently protected even by the partially degraded c-*myc* mRNA. With the exception of the H4 neuroglioma cell lines, all the cell lines yielded strong vimentin signals, while GFAP mRNA was detectable only in U-373, U-251 MG, U-251 MG-Sp and U-178 MG cells. None of the cell lines contained NF-M mRNA as analysed by Northern hybridisation. These expression data are summarised in Table III. As in the tumours, *max* mRNA processing patterns exhibited cell line-specific variation, resulting in the production of Max and ΔMax-encoding mRNAs at varying proportions. Data on these alternative *max* mRNA splice variants are summarised in Table II.

### *Localisation of* myc *and GFAP mRNAs by* in situ *hybridisation*

In selected gliomas, *in situ* hybridisation was utilised to localise and identify cells expressing *myc* and GFAP genes. Great topographic variation was observed in the *myc* hybridisation signal intensities. The *myc* autoradiographic grains were localised over malignant cells, but the strongest signals did not co-localise with regions of the highest mitotic activity. The glioblastoma with the highest c-*myc* mRNA levels (no. 10) displayed accentuated c-*myc* signals over some but not all malignant cell clusters, and in particular over pseudopalisadic tumour cell formations surrounding the necrotic lesion. In contrast, the reactive endothelial cell proliferations lacked a c-*myc* signal (Figure 5a–d). A few glioma cells also reacted with the N-*myc* probe, but the signal intensities were much weaker than with the c-*myc* probe. This weak N-*myc* signal was further confirmed by RNAase protections (see above: compare Figures 1 and 2). We observed an inverse topographic localisation of the GFAP and c-*myc* hybridisation signals: the cells with the highest c-*myc* expression showed only a weak or no GFAP signal (compare Figure 5b and d), while strongly GFAP-positive cells lacked c-*myc* mRNA (compare Figure 5f and h).

The SCGB tumour (no. 13) which had very high N-*myc* mRNA levels, displayed a non-identical topographic distribution of N-*myc*, L-*myc* and c-*myc* mRNA signals. The tumour cells displayed heterogeneous N-*myc* signal with some very intensely labelled tumour cell clusters, which lacked c/L-*myc* RNA (Figure 6a–e). In contrast, within areas with the highest proliferative activity only scattered cells contained N-*myc* mRNA. Areas containing reactive astrocytes mixed among malignant cells yielded positive hybridisation signals with c-*myc*, N-*myc* and L-*myc* probes, but not with entirely similar distribution patterns (Figure 6f–j). Thus, subpopulations of cells with differentially activated *myc* expression exist within this rare tumour.

### Discussion

Several glioma tumours contained N-*myc* and L-*myc* mRNAs as detected by RNAase protection, in addition to the high c-*myc* mRNA levels present in most glioma tumours and cell lines. Some tumours and most cell lines exhibited strong *max* mRNA signals, with evidence of differential alternative splicing. *RB1* mRNA was detected in all the gliomas, suggesting that *RB1* deletions play no role in glial cell transformation. However, subtle mutations abolishing the production of a normal RB1 protein cannot be detected by the methods used. Vimentin mRNA was detectable in all the tumours and cell lines, GFAP in most glioma tumours but in only four glioma cell lines. NF-M mRNA was absent from the cell lines and present in only a few low-grade gliomas, the signals most probably originating from neurons within the tumour tissue. No definite clinical characteristics could be assigned to different *myc* or *max* expression profiles in this limited set of patients. However, the higher c-*myc* mRNA levels and simultaneous presence of N-*myc* and L-*myc* mRNAs paralleled a higher grade of malignancy in astrocytomas grade I–III, whereas in the grade IV classic glioblastomas N-*myc* and L-*myc* were not active. The enhanced *myc* expression probably results from increased transcription or prolonged

**Figure 5** *In situ* hybridisation analysis of c-*myc*, N-*myc* and GFAP mRNA. **a**–**d** and **e**–**h**, respectively, represent the same fields of serial sections of a glioblastoma (tumour no. 10) hybridised with the different probes. **a**–**d**, A strong c-*myc* signal is seen in **b** over malignant cells surrounding the necrotic area (n) and the endothelial cell proliferation (en), while most c-*myc*-positive cells remain GFAP negative (**d**). Some c-*myc*-reactive cells also yield a weak N-*myc* signal (**c**). This N-*myc* signal was verified by RNAase protection analyses (Figure 2), although in Northern hybridisation this tumour appeared devoid of N-*myc* mRNA. **e**–**h**, An opposite topographic distribution is evident for the c-*myc* *vs* GFAP signals. The reactive astrocytes in this field show a very strong GFAP signal (**h**), only an extremely weak c-*myc* signal (**f**), and no N-*myc* signal (**g**). Bar = 100 μm.

mRNA half-life, since we did not detect amplified *myc* genes in our tumour panel as analysed by Southern hybridisation.

Enhanced expression of the N-*myc* gene has been implicated in the pathogenesis of extracranial tumours with neuroectodermal characteristics, such as peripheral neuroblastomas and some cases of SCLC (DePinho *et al.*, 1991). In neuroblastomas, N-*myc* amplification is a strong indicator of poor prognosis, independently of other staging criteria (Brodeur *et al.*, 1984; Seeger *et al.*, 1985). Our results show that some glioma tumours may express N-*myc* mRNA simultaneously with c-*myc* and/or L-*myc*. The N-*myc* mRNA levels in these tumours are detectable by RNAase protection but not by Northern hybridisation. The partially overlapping but non-identical topographic distribution patterns of the *myc in situ* hybridisation signals are suggestive of differential regulation of the genes in glial cells. The high levels of N-*myc* in the SCGB differed sharply from other high-grade gliomas. The histopathological picture of this tumour was dominated by

**Figure 6** Differential localisation of *myc* mRNAs in the small-cell glioblastoma tumour (SCGB). **b**, A strong N-*myc* signal in tumour cells that lack c-*myc* (**c**), L-*myc* (**d**) and GFAP (**e**) mRNAs. **a**, A bright-field image of the same field shown in **b**–**e**. **f**–**j**, Tumour cells mixed among few reactive astrocytes show scattered, but not strictly identical distribution, patterns of c-*myc* (**h**), N-*myc* (**g**) and L-*myc* (**i**) mRNAs, but lack of GFAP mRNA (**j**). Bar in **a** (for **a**–**e**) and **f** (for **f**–**j**) = 200 μm.

undifferentiated small cells identical to those characteristic of primitive neuroecotodermal tumours (PNETs). This, together with the presence of NF-M and vimentin mRNA and the lack of GFAP mRNA, makes it difficult to assign a specific diagnosis for this tumour. The diagnosis of PNET with neuronal and glial differentiation would be justified for this rare tumour, but since PNET terminology is somewhat controversial the diagnosis of SCGB has been adhered to here.

The finding prompts further investigations as to whether high-level N-*myc* expression could be a useful marker for malignant small-cell tumours in the brain, i.e. for the disputed PNET group (Rorke, 1983), particularly if complemented with other cell type-specific markers of tumour differentiation and stage.

L-*myc* expression has not been previously reported in gliomas. In our tumour material, several gliomas contained L-*myc* mRNAs at levels equal to or in excess of those in fetal brain. This underlines the usefulness of sensitive RNAase protection analyses rather than ordinary Northern hybridisation in studies of low-abundancy mRNAs like L-*myc*. Even though L-*myc* (and N-*myc*) mRNAs were present in most grade III malignant astrocytomas, they were absent from two classic glioblastomas. In contrast, the glioma cell lines adapted to growth *in vitro* lacked L-*myc* and N-*myc* mRNAs, but most of them contained high-levels of c-*myc* mRNA. This finding is consistent with earlier observations of a lack of N-*myc* and L-*myc* mRNAs and presence of c-*myc* mRNA in glioma cell lines (LaRocca *et al.*, 1989).

*In situ* hybridisations revealed the highest *myc* mRNA levels in regions distinct from those with the highest proliferative activity. This finding parallels our observations and those of others of uncoupling of N-*myc* (Grady *et al.*, 1987; Hirvonen *et al.*, 1989) as well as c-*myc* and L-*myc* (Hirvonen *et al.*, 1990) expression from mitotic activity in fetal brain. In gliomas, the high *myc* mRNA levels may be linked to a dedifferentiated state rather than proliferative activity, as is the case in developing fetal CNS. This notion is supported by the finding that GFAP and c-*myc* mRNAs display mutually exclusive topographic distribution patterns in gliomas as analysed by *in situ* hybridisation. Similarly, the glioma cell lines with the highest c-*myc* expression levels were negative for GFAP mRNA but did contain vimentin mRNA, which is the earlier of the two intermediate filaments expressed during glial cell differentiation.

Several glioma cells and tumours contained *max* mRNA at levels equal to or in excess of those found in normal human fetal brain. *max* mRNA levels in glioma cells are regulated independently of the *myc* genes, and show great variation between different cell lines. Interestingly, the *max* mRNA processing patterns varied between different glioma tumours and glioma lines. This may be of functional importance, since alternative *max* mRNA processing results in at least five different mRNA forms with different protein-coding capacities and opposing effects on the transforming activity of Myc. The first identified, Max (Blackwood & Eisenman, 1991), suppresses while the C-terminally truncated ΔMax augments Myc transformation (Mäkelä *et al.*, 1992). In addition to the major 2.3- and 2.4-kb bands representing Max- and ΔMax-encoding mRNAs, respectively, several glioma cells contained additional *max* mRNAs of 1.9 kb, 3.0 kb and 3.5 kb. Recent data indicate that the 3.0-kb and 3.5-kb *max* mRNAs encode Max polypeptides that are structurally identical (3.0 kb) or highly similar (3.5 kb) to ΔMax, and which enhance the transforming activity of Myc in Myc–Ras co-transformation assays (Västrik *et al.*, 1993). The amount and composition of the different Myc–Max heterodimers may thus be regulated via *max* transcription and mRNA splicing, in addition to the regulation of *myc* mRNA levels. In K562 cells, a pH decrease causes a switch from the 2.3- and 2.4-kb *max* mRNAs to the 3.5-kb mRNA form, suggesting that acidification of the culture medium may regulate *max* mRNA processing (Västrik *et al.*, 1993). In the case of the glioma cells analysed here we consider this an unlikely source of *max* mRNA splice pattern variation between different cell lines, as all the cultures were confluent and medium was changed 24 h prior to RNA extraction. Further studies will undoubtedly clarify the functional significance of the different Max forms in normal and malignant neuroectodermal cells and their derivatives.

We have previously reported combinatorial expression of several *myc* genes in human leukaemias and leukaemia cell lines (Hirvonen *et al.*, 1991). Our present results suggest that some gliomas may coexpress several *myc* genes, thus in part augmenting the restricted tissue spectrum of N-*myc* and L-*myc* expression. It is possible that L-*myc* and N-*myc* are transiently activated in some early stages of the transformation of glial cells and contribute to their escape from their growth restraints. In this context it is interesting that sustained N-*myc* expression in neuroblastoma cells maintains their phenotypic instability manifested as a transdifferentiation potential, which can be abolished by blocking N-Myc production using antisense N-*myc* expression constructs (Whitesell *et al.*, 1991). The discrepancy between L-*myc* and N-*myc* expression in gliomas *in vivo* as opposed to glioma cells *in vitro* does not result from eventual normal cells trapped within the biopsy specimens, as in the *in situ* analyses the L-*myc* and N-*myc* signals were located over neoplastic glial cells. It is possible that culture conditions select for the glioma cells with the highest c-*myc* expression levels, which conceivably would result in a cell population with silent N-*myc* and L-*myc* genes, as a result of negative *myc* cross-regulation. Since c-*myc* but not N- or L-*myc* appears to be involved in the signalling pathways of several growth factors, c-*myc* expression might best allow the glioma cells to circumvent the lack of exogenous growth factors in culture. Further analysis of *myc* and *max* in normal CNS cells and identification of their target genes in glioma cells will undoubtedly be helpful in establishing their roles in normal and malignant glial cell growth.

Professor Karl Alitalo is gratefully acknowledged for valuable discussions and critical reading of the manuscript, Dr Tomi Mäkelä for the *max* cDNA clone, Professor Bengt Westermark for the glioma cell lines and Mrs Tuula Oivanen, Mrs Merja Lakkisto and Mrs Liisa Lempiäinen for expert technical assistance. These studies were financially supported by the Academy of Finland, The Finnish Cancer Foundation, the Cultural Foundation of Southwest Finland, the Jusélius Foundation and the Farmos and Orion Research Funds.

### References

ALITALO, K., KOSKINEN, P., MÄKELÄ, T.P., SAKSELA, K., SISTONEN, L. & WINQVIST, R. (1987). *myc* oncogenes: activation and amplification. *Biochim. Biophys. Acta*, **907**, 1–32.

BIGNER, S.H., BURGER, P.C., WONG, A.J., WERNER, M.H., HAMILTON, S.R., MUHLBAIER, L.H., VOGELSTEIN, B. & BIGNER, D.D. (1988). Gene amplification in malignant human gliomas: clinical and histopathologic aspects. *J. Neuropathol. Exp. Neurol.*, **47**, 191–205.

BLACKWOOD, E.M. & EISENMAN, R.N. (1991). Max: a helix–loop–helix zipper protein that forms a sequence-specific DNA-binding complex with Myc. *Science*, **251**, 1211–1217.

BRODEUR, G.M., SEEGER, R.C., SCHWAB, M., VARMUS, H.E. & BISHOP, J.M. (1984). Amplification of N-*myc* in untreated human neuroblastomas correlates with advanced disease state. *Science*, **224**, 1121–1124.

CHIRGWIN, J.M., PRZYBYLA, A.E., MACDONALD, R.J. & RUTTER, W.J. (1979). Isolation of biologically active ribonucleic acid from sources enriched in ribonuclease. *Biochemistry*, **18**, 5294–5299.

DAUMAS-DUPORT, C., SCHEITHAUER, B., O'FALLON, J. & KELLY, P. (1988). Grading of astrocytomas. A simple and reproducible method. *Cancer*, **62**, 2152–2165.

DEPINHO, R.A., SCHREIBER-AGUS, N. & ALT, F.W. (1991). *myc* family oncogenes in the development of normal and neoplastic cells. *Adv. Cancer Res.*, **57**, 1–46.

FEINBERG, A.P. & VOGELSTEIN, B. (1983). A technique for radiolabeling DNA restriction endonuclease fragments to high specific activity. *Anal. Biochem.*, **132**, 6–13.

FUJIMOTO, M., SHERIDAN, P.J., SHARP, Z.D., WEAKER, F.J., KAGAN-HALLET, S. & STORY, J.L. (1989). Proto-oncogene analyses in brain tumors. *J. Neurosurg.*, **70**, 910–915.

GALLIE, B.L., SQUIRE, J.A., GODDARD, A., DUNN, J.M., CANTON, M., HINTON, D., ZHU, X. & PHILLIPS, R.A. (1990). Mechanism of oncogenesis in retinoblastoma. *Lab. Invest.*, **62**, 394–408.

GRADY, E.F., SCHWAB, M. & ROSENAU, W. (1987). Expression of N-*myc* and c-*src* during the development of fetal human brain. *Cancer Res.*, **47**, 2931–2936.

HIRVONEN, H., SANDBERG, M., KALIMO, H., HUKKANEN, V., VUORIO, E., SALMI, T.T. & ALITALO, K. (1989). The N-*myc* proto-oncogene and IGF-II growth factor mRNAs are expressed by distinct cells in human fetal kidney and brain. *J. Cell Biol.*, **108**, 1093–1104.

HIRVONEN, H., MÄKELÄ, T.P., SANDBERG, M., KALIMO, H., VUORIO, E. & ALITALO, K. (1990). Expression of the *myc* proto-oncogenes in human fetal brain development. *Oncogene*, **5**, 1787–1797.

HIRVONEN, H., HUKKANEN, V., SALMI, T.T., MÄKELÄ, T.P., PELLINIEMI, T.-T., KNUUTILA, S. & ALITALO, R. (1991). Expression of L-*myc* and N-*myc* proto-oncogenes in human leukemias and leukemia cell lines. *Blood*, **78**, 3012–3020.

JAMES, C.D. & COLLINS, V.P. (1992). Molecular genetic characterization of CNS tumor oncogenesis. *Adv. Cancer Res.*, **58**, 121–142.

JOHNSON, B.E, MAKUCH, R.W., SIMMONS, A.D., GAZDAR, A.F., BURCH, D. & CASHELL, A.W. (1988). *myc* family DNA amplification in small cell lung cancer patients' tumors and corresponding cell lines. *Cancer Res.*, **48**, 5163–5166.

KAYE, F., BATTEY, J., NAU, M., BROOKS, B., SEIFTER, E., DE GREVE, J., BIRRER, M., SAUSVILLE, E. & MINNA, J. (1988). Structure and expression of the human L-*myc* gene reveal a complex pattern of alternative mRNA processing. *Mol. Cell. Biol.*, **8**, 186–195.

KOHL, N.E., GEE, C.E. & ALT, F.W. (1984). Activated expression of the N-*myc* gene in human neuroblastomas and related tumors. *Science*, **226**, 1335–1337.

LAROCCA, R.V., ROSENBLUM, M., WESTERMARK, B. & ISRAEL, M.A. (1989). Patterns of proto-oncogene expression in human glioma cell lines. *J. Neurosci. Res.*, **24**, 97–106.

LIBERMANN, T.A., NUSSBAUM, H.R., RAZON, N., KRIS, R., LAX, I., SOREQ, H., WHITTLE, N., WATERFIELD, M.D., ULLRICH, A. & SCHLESSINGER, J. (1985). Amplification, enhanced expression and possible rearrangement of EGF receptor gene in primary human brain tumors of glial origin. *Nature*, **313**, 144–147.

LÜSCHER, B. & EISENMAN, R.N. (1990). New light on Myc and Myb. I. Myc. *Genes Dev.*, **4**, 2025–2035.

MÄKELÄ, T.P., KOSKINEN, P., VÄSTRIK, I. & ALITALO, K. (1992). Alternative forms of Max can act either as enhancers or suppressors of cotransformation by c-Myc and Ras. *Science*, **256**, 373–377.

MUKHERJEE, B., MORGENBESSER, S.D. & DEPHINHO, R.A. (1992). Myc family oncoproteins function through a common pathway to transform normal cells in culture: cross-interference by Max and *trans*-acting dominant mutants. *Genes Dev.*, **6**, 1480–1492.

MYERS, M., LAZZARINI, R.A., LEE, V.M.-Y., SCHLAEPFER, W.W. & NELSON, D.L. (1987). The human mid-size neurofilament subunit: a repeated protein sequence and the relationship of its gene to the intermediate filament gene family. *EMBO J.*, **6**, 1617–1626.

NAU, M.M., BROOKS, D.N., BATTEY, J., SAUSVILLE, E., GAZDAR, A.F., KIRSCH, I.R., MCBRIDE, O.W., BERTNESS, V. & HOLLIS, G.F. (1985). L-*myc*, a new *myc*-related gene amplified and expressed in human small cell lung cancer. *Nature*, **318**, 69–73.

NAU, M.M., BROOKS, B.J., CARNEY, D.N., GAZDAR, A.F., BATTEY, J.F., SAUSVILLE, E.A. & MINNA, J.D. (1986). Human small-cell lung cancers show amplification and expression of the N-*myc* gene. *Proc. Natl Acad. Sci. USA*, **83**, 1092–1096.

NISHIYAMA, A., ONDA, K., WASHIYAMA, K., KUMANISHI, T., KUWANO, R., SAKIMURA, K. & TAKAHASHI, Y. (1989). Differential expression of glial fibrillary acidic protein in human glioma cell lines. *Acta Neuropathol.*, **78**, 9–15.

ÖFVERSTEDT, L.-G., HAMMARSTRÖM, K., BALGOBIN, N., HJERTEN, S., PETTERSON, U. & CHATTOPADHYAYA, J. (1984). Rapid and quantitative recovery of DNA fragments from gels by displacement electrophoresis (isotachophoresis). *Biochim. Biophys. Acta*, **782**, 120–126.

PERREAU, J., LILIEANBAUM, A., VASSEUR, M. & PAULIN, D. (1988). Nucleotide sequence of the human vimentin gene and regulation of its transcription in tissues and cultured cells. *Gene*, **62**, 7–16.

PRENDERGAST, G.C., LAWE, D. & ZIFF, E.B. (1991). Association of Myn, the murine homolog of Max, with c-Myc stimulates methylation-sensitive DNA binding and Ras cotransformation. *Cell*, **65**, 395–407.

RORKE, L.B. (1983). The cerebellar medulloblastoma and its relationship to primitive neuroectodermal tumors. *J. Neuropathol. Exp. Neurol.*, **42**, 1–15.

RUSTGI, A.K., DYSON, N. & BERNARDS, R. (1991). Amino-terminal domains of c-*myc* and N-*myc* proteins mediate binding to the retinoblastoma gene product. *Nature*, **352**, 541–544.

SAKSELA, K., MÄKELÄ, T. & ALITALO, K. (1989). Oncogene expression in small-cell lung cancer cell lines and a testicular germ cell tumor: activation of the N-*myc* gene and decreased RB mRNA. *Int. J. Cancer*, **44**, 182–185.

SANDBERG, M. & VUORIO, E. (1987). Localization of types I, II and III collagen mRNAs in developing human skeletal tissues by *in situ* hybridization. *J. Cell Biol.*, **104**, 1077–1084.

SCHWAB, M., ELLISON, J., BUSCH, M., ROSENAU, W., VARMUS, H.E. & BISHOP, J.M. (1984). Enhanced expression of the human N-*myc* consequent to amplification of DNA may contribute to malignant progression of neuroblastoma. *Proc. Natl Acad. Sci. USA*, **81**, 4940–4944.

SEEGER, R.C., BRODEUR, G.M., SATHER, H., DALTON, A., SIEGEL, S.E., WONG, K.Y. & HAMMOND, D. (1985). Association of multiple copies of the N-*myc* oncogene with rapid progression of neuroblastomas. *N. Engl. J. Med.*, **313**, 1111–1116.

TRENT, J., MELTZER, P., ROSENBLUM, M., HARSH, G., KINZLER, K., MASHAL, R., FEINBERG, A. & VOGELSTEIN, B. (1986). Evidence for rearrangement, amplification, and expression of c-*myc* in a human glioblastoma. *Proc. Natl Acad. Sci. USA*, **83**, 470–473.

VÄSTRIK, I., KOSKINEN, P.J., ALITALO, R. & MÄKELÄ, T.P. (1993). Alternative mRNA forms and open reading frames of the *max* gene. *Oncogene*, **8**, 503–507.

WELTER, C., HENN, W., THEISINGER, B., FISCHER, H., ZANG, K.D. & BLIN, N. (1990). The cellular *myb* oncogene is amplified rearranged and activated in human glioblastoma cell lines. *Cancer Lett.*, **52**, 57–62.

WENZEL, A., CZIEPLUCH, C., HAMANN, U., SCHÜRMANN, J. & SCHWAB, M. (1991). The N-*myc* oncoprotein is associated *in vivo* with the phosphoprotein Max(p20/22) in human neuroblastoma cells. *EMBO J.*, **10**, 3703–3712.

WHITESELL, L., ROSOLEN, A. & NECKERS, L.M. (1991). Antisense suppression of N-*myc* expression inhibits the transdifferentiation of neuroectoderm tumor cell lines. *Prog. Clin. Biol. Res.*, **366**, 45–54.

WONG, A.J., RUPPERT, J.M., EGGLESTON, J., HAMILTON, S.R., BAYLIN, S.B. & VOGELSTEIN, B. (1986). Gene amplification of c-*myc* and N-*myc* in small cell carcinoma of the lung. *Science*, **233**, 461–464.

ZÜLCH, K.J. (1979). *International Histological Classification of Tumours* No. 21. *Histological Typing of Tumours of the Central Nervous System*. Geneva: World Health Organization.